# Integrated Transcriptome and Targeted Metabolite Analysis Reveal miRNA-mRNA Networks in Low-Light-Induced Lotus Flower Bud Abortion

**DOI:** 10.3390/ijms23179925

**Published:** 2022-09-01

**Authors:** Huihui Ren, Yingchun Xu, Hongsheng Lixie, Jiaying Kuang, Yanjie Wang, Qijiang Jin

**Affiliations:** Key Laboratory of Landscaping, Ministry of Agriculture and Rural Affairs, Key Laboratory of Biology of Ornamental Plants in East China, College of Horticulture, Nanjing Agricultural University, Nanjing 210095, China

**Keywords:** *Nelumbo nucifera*, flower bud abortion, transcriptome, metabolite

## Abstract

Most *Nelumbo nucifera* (lotus) flower buds were aborted during the growing season, notably in low-light environments. How lotus produces so many aborted flower buds is largely unknown. An integrated transcriptome and targeted metabolite analysis was performed to reveal the genetic regulatory networks underlying lotus flower bud abortion. A total of 233 miRNAs and 25,351 genes were identified in lotus flower buds, including 68 novel miRNAs and 1108 novel genes. Further enrichment analysis indicated that sugar signaling plays a potential central role in regulating lotus flower bud abortion. Targeted metabolite analysis showed that trehalose levels declined the most in the aborting flower buds. A potential regulatory network centered on miR156 governs lotus flower bud abortion, involving multiple miRNA-mRNA pairs related to cell integrity, cell proliferation and expansion, and DNA repair. Genetic analysis showed that miRNA156-5p-overexpressing lotus showed aggravated flower bud abortion phenotypes. Trehalose-6-P synthase 1 (TPS1), which is required for trehalose synthase, had a negative regulatory effect on miR156 expression. *TPS1*-overexpression lotus showed significantly decreased flower bud abortion rates both in normal-light and low-light environments. Our study establishes a possible genetic basis for how lotus produces so many aborted flower buds, facilitating genetic improvement of lotus’ shade tolerance.

## 1. Introduction

With the acceleration of urbanization, many green spaces are surrounded by buildings, and more than 50% of green spaces are in hidden environments, which indicates that many plants cannot get enough sunlight to grow and develop normally, due to being shaded by trees, buildings, and other structures in urban areas. Long-term insufficient light not only greatly limits the growth of plants, but also restricts their growing regions and ornamental value. In addition, insufficient indoor light also limits the indoor green area.

Light is the source of energy for plants, as it drives photosynthesis to produce sugars which play a crucial role in flower bud differentiation [1,2]. Inversely, shading induces flower bud abscission by affecting source strength [3,4]. As in sweet pepper, shading for short periods or leaf pruning weakens the source strength and easily causes flower bud abortion during the first week after anthesis [5]. Similarly, additional light was needed to prevent flower bud abscission and flower bud blasting in *Asiatic lilies* during the winter period [6]. It is clearly indicated that the reduction in carbohydrate transportation to flower buds by low light leads to flower bud abortion and sugar remains the scarcest resource in flower buds even under the most suitable growth conditions [7].

Sugars not only provide energy for metabolic processes, but also act as signaling molecules to modulate the expression of a series of small RNAs, transcription factors, and other stress resistance genes, such as sucrose non-fermenting-1-related protein kinase 1 (SnRK1) and target of rapamycin (TOR) [8,9,10]. Trehalose-6-phosphate (T6P), a metabolic intermediate of the trehalose biosynthetic pathway, has been shown to be an important signaling molecule that measures sugar status and regulates a wide range of developmental processes [11]. MicroRNAs (miRNAs) are noncoding 21–22 nucleotide (nt) RNAs [12,13]. At present, a handful of miRNAs and their target genes have been found to be implicated in diverse stages of plant flowering [14,15]. As one of the highly conserved miRNAs, miR156, along with its target gene, the SQUAMOSA-promoter binding-like (SPL) gene family, plays an important role in plant growth and development [16,17], metabolic regulation [18], and abiotic stress responses [19]. In *Arabidopsis thaliana*, Squamosa promoter-binding protein-like 3 (SPL3), Squamosa promoter-binding protein-like 4 (SPL4) and Squamosa promoter-binding protein-like 5 (SPL5) can promote nutrient phase transition and flowering and are strongly inhibited by miR156 [20]. The studies of Olas, et al. [21] and Wahl, et al. [22] suggested that the T6P pathway controls expression of SPL3, SPL4, and SPL5 in shoot apical meristems (SAM), partly via miR156 and partly independently of the miR156-dependent age pathway, in *Arabidopsis thaliana*. MiR156 is likely to act as a “scale” in plants to sense carbohydrate availability in the body and further regulate the flowering process.

Lotus (*Nelumbo nucifera* Gaertn) is a perennial aquatic ornamental plant mainly cultivated for flowers, edible rhizomes, seeds, and medicinal purposes [23,24]. The industrialized production of lotus and its application in garden landscapes are also very common. However, the lotus is very light-loving and requires full exposure to the sun during the growth period. Our previous study reported a close relationship between flower buds and weather conditions, revealing the eventual development of lotus flower buds under strong light on sunny days and vice versa under low light in rainy conditions [24]. Most flower buds were aborted under harsh environmental conditions, specifically in low-light environments, which greatly affects its growing regions and ornamental value [25]. However, how lotus produced so many aborted flower buds remains unknown. In this study, we conducted integrated transcriptomic and miRNA analyses of aborting flower buds caused by low light, combined with carbohydrate metabolite analysis of normal and aborting flower buds, trying to find the key points behind and establish the potential transcriptomic and genetic regulatory networks of low-light-induced lotus flower bud abortion. 

## 2. Results

### 2.1. High-Throughput Sequencing of Small RNAs and mRNAs in Aborting Lotus Flower Buds

To explore the changes of small RNAs and mRNAs between the aborting (Ab) and normal flower buds (Ck), six small RNA libraries and six mRNA libraries were separately constructed and sequenced. Overviews of the sequencing and assembly results are listed in Table S1. A total of six libraries were sequenced using the BGISEQ-500 platform for mRNA transcriptomes, and each sample generated 6.40 GB of sequence data on average. The average alignment rate was 92.88% for the genome and 81.45% for gene sets. mRNA sequencing identified 25,138 genes including 24,105 known genes and 1033 novel genes. A total of 10,863 new transcripts were detected, of which 7276 belong to new alternative splicing subtypes of known protein-coding genes, 1123 belong to new protein-coding transcripts, and the remaining 2464 belong to long-chain non-coding RNA. We obtained 13.11 Gb of transcriptome data from small RNA sequencing using the BGISEQ-500 platform. After filtering, clean tags of the sRNA libraries were mapped to the sRNA database including miRbase, Rfam, siRNA, piRNA, and snoRNA (Table S2). It is well accepted that 21–24 nt is the common length of functional small RNAs. The sequence length distribution of sRNAs has no obvious difference among the six samples. The length of the cleanest reads ranged from 21 to 24 nt, with the 24 nt sRNA being the most abundant, followed by 21 nt (Figure S1). We identified 165 known miRNAs representing 37 families and 68 novel miRNAs. Most of the identified known miRNAs (56.35%) belong to the 21 nt-length miRNA families, followed by the 20 nt (24.24%)-length miRNA families (Figure S2). In contrast, the most abundant (16.18%) novel miRNAs were 30 nt in length, followed by 28 nt (13.24%, Figure S2). Overall, the miR396 family has the most abundant members in lotus, followed by miR156, miR171, miR166, miR172, miR169, and miR319. For the four nucleic acids, the frequency of adenosine (A), uracil (U), cytosine (C), and guanine (G) accounted for 23.52%, 25.55%, 24.59%, 26.34% (Figure S2), respectively. The first nucleotide bias analysis showed that G and A had the absolute predominance in miRNAs. The length of the 68 novel miRNAs ranges from 19 to 30 nt.

### 2.2. Differentially Expressed miRNAs and Genes in the Normal and Aborting Flower Buds

**Figure 1:**
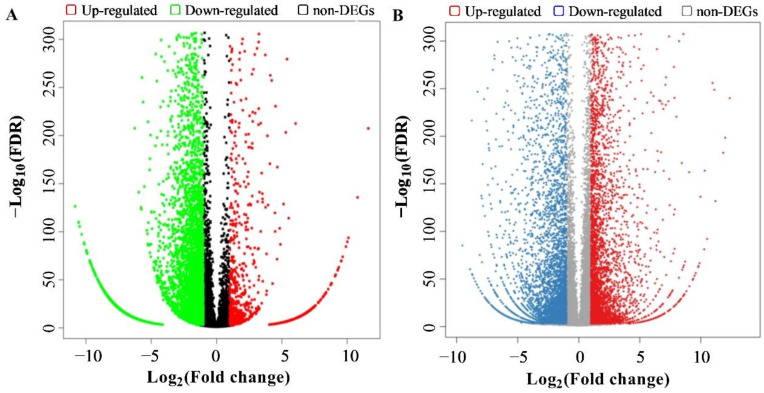
High-throughput small RNA and mRNA sequencing revealed abortion-related miRNA and genes in lotus flower buds. Volcano plots of differentially expressed small RNAs (**A**) and genes (**B**) between normal and aborting flower buds.

MicroRNAs (miRNAs) are critical regulators of gene expression. Distinctive expression patterns of miRNAs between Ab and Ck libraries provided an opportunity to identify important miRNAs related to flower bud abortion. A total of 182 miRNAs were differentially expressed (Table S3). Among them, 149 miRNAs including 86 up-regulated and 63 down-regulated miRNAs were known miRNAs which belonged to 36 different families. The abundance of the 149 known miRNAs showed large differences, with the Fragments Per Kilobase Million (FPKM) value varying from 0 to 361,162. miR166 showed the highest transcript abundance in both libraries, followed by miR159, miR858, miR171, miR168, and miR167. A total of 33 novel miRNAs including 20 up-regulated and 13 down-regulated ones were differentially expressed. We found 66 significantly changed miRNAs using the stringent cutoffs fold change ≥1, *p*-value ≤ 0.05, and Q ≤ 0.001 (Table S3), including 36 up-regulated and 30 down-regulated miRNA (Figure 1). The log_2_FoldChange (Ab/Ck) of gene expression levels was in the range of −6.63 to 7.95. Specifically, novel_mir47 was the most up-regulated miRNA, followed by miR319a-3p, novel_mir31, miR398, miR398b, and novel_miR11 with fold changes of 7.47, 6.14, 6.10, 5.78, and 5.68, respectively. In contrast, miR159b-5p was the most clearly down-regulated miRNA, followed by miR319a-5p_1, miR395a_5, miR319b-5p_2, miR169f.1, and miR169e_3 with fold changes of −5.99, −5.23, −4.91, −4.08, and −3.37, respectively. Same-family members often showed similar expression patterns, such as for miR166, miR167, miR168, miR395, miR398, and miR408.

In the transcriptome data, we found major differences in gene expression when comparing normal and aborting flower buds (Table S4). In total, 5252 genes were differentially expressed with log_2_ fold change ≥2 and *p* value ≤ 0.05. Of these, 2211 genes were up-regulated and 3041 were down-regulated in aborting flower buds compared with the normal flower buds (Ck). 

### 2.3. Identification and Functional Characterization of miRNA Potential Targets

In this study, 163 miRNAs including 153 conserved and 10 novel miRNAs were predicted to target 3076 potential target genes using different methods (Table S5). Some miRNAs have no predicted target gene, probably because of the limitations in available genome data or because they have no actual targets. A total of 750 potential target genes were predicted for 50 significantly differentially expressed miRNAs, including 43 conserved and 7 novel miRNAs (Table S5). The results showed that most miRNAs have more than one predicted target mRNA, i.e., miR156_2 has up to 148 predicted target mRNAs. In addition, one transcript could also be targeted by several miRNAs, for example, XM_010258909.2 was targeted by miR169b-5p, miR169e_3, miR169h_2, and miR169r-5p. Usually, mature miRNAs negatively regulate gene expression by inhibition of translation or target cleavage. Here, a total of 156 miRNA-mRNA pairs were found including 32 differentially expressed miRNAs and their target genes based on mRNA sequencing (Table S6). 

**Figure 2:**
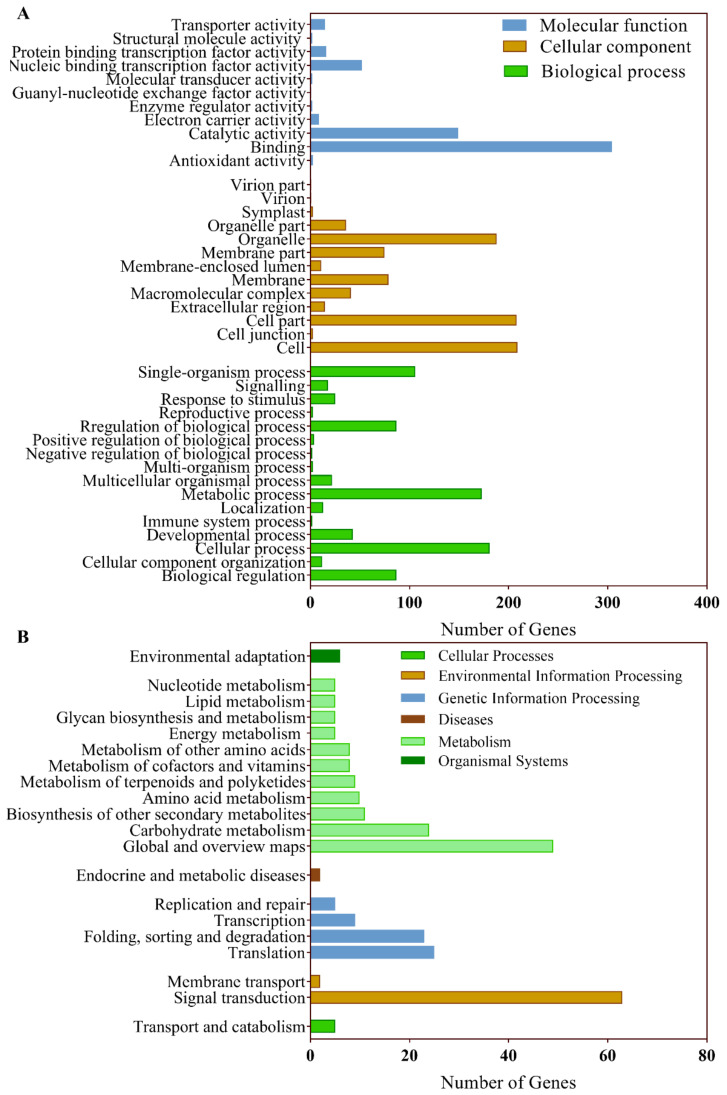
High-throughput small RNA and mRNA sequencing revealed abortion-related small RNAs and mRNAs in lotus flower buds. Volcano plots of differentially expressed small RNAs (**A**) and miRNAs (**B**) between the normal and aborting flower buds.

The functional classification of differentially expressed miRNA targets was performed with Gene Ontology (GO) terms and Kyoto Encyclopedia of Genes and Genomes (KEGG) pathway enrichment analysis with the aim to elucidate the biological processes involved in flower bud abortion (Figure 2A). The majority of GO terms assigned from the molecular function category were associated with binding (GO: 0005488; 76.2%) and catalytic activity (GO: 0003824; 37.3%). As for the cellular component, the major of GO terms were cell (GO: 0005623; 74.1%), cell part (GO: 0044464; 73.8%), and organelle (GO: 0043226; 66.7%). In terms of biological process, cellular process (GO: 0009987; 86.6%) is the most abundant GO annotation, followed by metabolic process (GO: 0008152; 74.1%).

Moreover, 279 target genes were annotated in 66 KEGG pathways (Figure 2B). According to the KEGG enrichment analysis, these genes are primarily associated with metabolism and signal transduction. The global and overview maps contained the largest number of differential expression genes (DEGs), followed by carbohydrate metabolism. In environmental information processing, many subterms associated with flowering, such as the pathways of plant hormone signal transduction (60 genes), were enriched. In particular, miR156′s target genes, the SPLs, dominated in these pathways, followed by miR167d_1′s target ARF6/8.

### 2.4. Expression Analysis of the Possible Causative miRNAs and Target Genes of the Abortion

**Figure 3:**
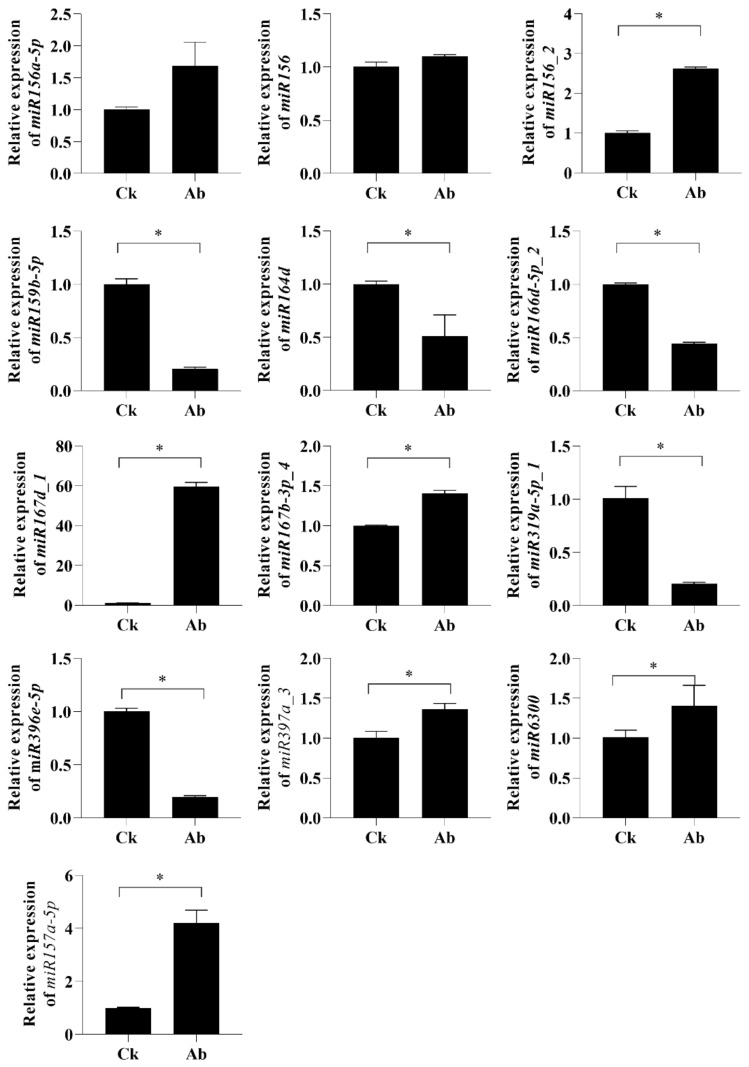
Relative expression level of 13 known miRNAs by RT-qPCR analysis. Normal (Ck) and aborting lotus flower buds (Ab) (2–3 cm in length) were collected for RT-qPCR analysis. Values are the means ± SE of three independent experiments with at least three replicates for each. U6 small nuclear RNA was used as an endogenous control to normalize the expression levels of miRNAs from RT-qPCR. Asterisks indicate that mean values are significantly different in comparison with Ck treatment at *p* < 0.05 according to a *t*-test. Ck, normal flower bud. Ab, aborting flower buds.

**Figure 4:**
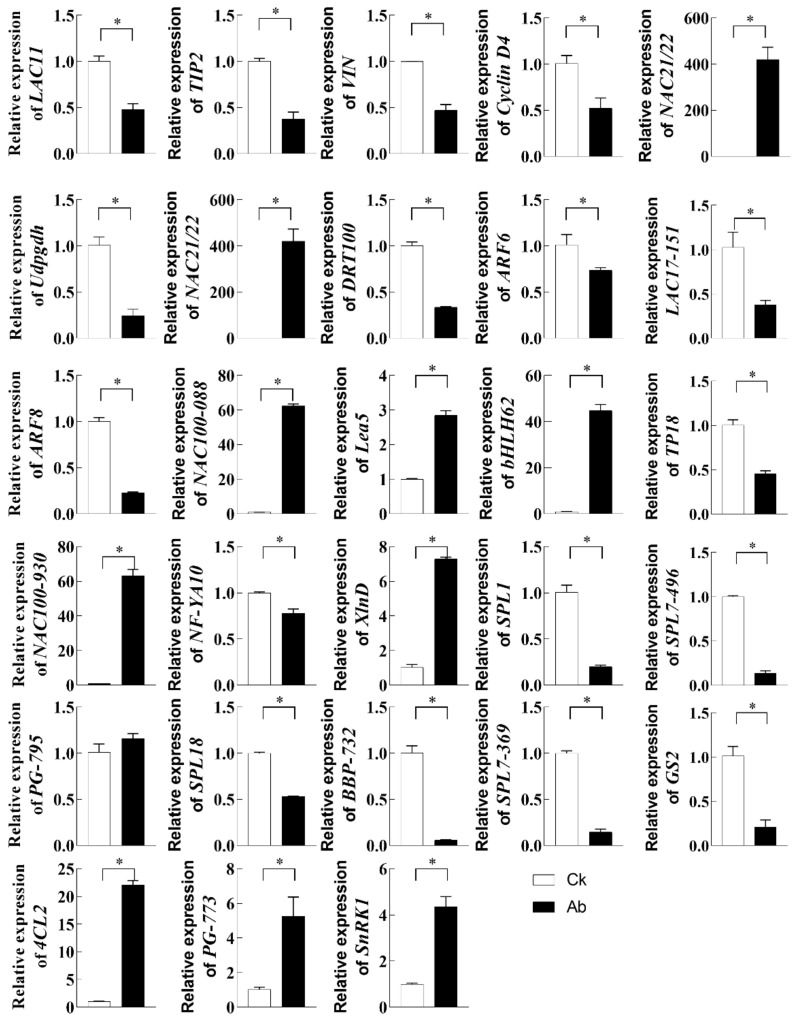
Relative expression level of 28 genes by RT-qPCR analysis. Normal (Ck) and aborting lotus flower buds (Ab) (2–3 cm in length) were collected for RT-qPCR analysis. Values are the means ± SE of three independent experiments with at least three replicates for each. Asterisks indicate that mean values are significantly different in comparison with Ck at *p* < 0.05 according to a *t*-test. *NnActin* (XM_010252745) were used for cDNA normalization. Udpgdh: UDP-glucose 6-dehydrogenase 5-like (LOC104598792); Cyclin-D6: Putative cyclin-D6-1 (LOC104605722); VIN: Beta-fructofuranosidase, soluble isoenzyme I-like (LOC104598577); Cyclin-D4: Cyclin-D4-1-like (LOC104613419); TIP2 (TIP2-1): Aquaporin Tonoplast Intrinsic Protein2-1 (LOC104595131); LAC11: Laccase-11-like (LOC104601843); NAC21/22: NAC domain-containing protein 21/22 (LOC104598350); DRT100: DNA-damage-repair/toleration protein DRT100-like (LOC104597475); ARF6: Auxin response factor 6 (LOC104610223); LAC17-151: Accase-17-like (LOC104597151); ARF8: Auxin response factor 8 (LOC104592798); NAC100-088: NAC domain-containing protein 100-like (LOC104598088); Lea5: Late embryogenesis abundant protein Lea5 (LOC104598918); bHLH62: Transcription factor bHLH62-like (LOC104587311); TP18: Transmembrane protein 18 (LOC104596748); NAC100-930: NAC domain-containing protein 100-like, (LOC104597930); NF-YA10: Nnuclear transcription factor Y subunit A-10-like (LOC104601425); XlnD: Probable beta-D-xylosidase 6 (LOC104595566); SPL1: Squamosa promoter-binding protein 1 (LOC104599433); SPL7-496: Squamosa promoter-binding-like protein 7 (LOC104598496); PG-795: Polygalacturonase-like (LOC104586795); SPL18: Squamosa promoter-binding-like protein 18 (LOC104591759); BBP-732: Basic blue protein-like (LOC104606732); SPL7-369: Squamosa promoter-binding-like protein (LOC104602369); GS2: Galactinol synthase 2-like (LOC104611225); 4CL2: 4-coumarate--CoA ligase 2-like (LOC104611494); PG-773: Polygalacturonase-like (LOC104608773); SnRK1: Sucrose non-fermenting-1-related protein kinase 1 (LOC104598048).

To validate the reliability of deep sequencing, we used qRT-PCR to analyze the expression of miRNAs (Figure 3) and corresponding target genes (Figure 4) that might lead to flower bud abortion.

MiRNAs exhibiting differential expression with at least a 2.0-fold change and corresponding target genes with opposite expression (fold change ≥1.5) and enriched in abortion-related GO terms or KEGG pathways were selected for RT-qPCR analysis. Overall, the expression patterns of these selected miRNAs obtained by qRT-PCR were consistent with the results from sRNA high-throughput sequencing (Figure 3). qRT-PCR was also performed to validate the expression data generated by mRNA sequencing, which showed a high consistency with the result of mRNA sequencing (Figure 4). The results also revealed that the predicted target genes had opposite expression profiles compared to the corresponding miRNAs. These results suggested that the sequencing data produced in the present study and the computational prediction of target genes were reliable.

### 2.5. Comparison of Carbohydrate Metabolites in the Normal and Aborting Flower Buds

Data from RNA deep sequencing revealed the important role of carbohydrate metabolites in regulating lotus flower bud abortion. Therefore, we carried out targeted metabolite analysis to obtain quantitative information on carbohydrates in the normal and aborting flower buds. In total, 13 sugars including 9 monosaccharides and 4 disaccharides were identified in the normal and/or aborting flower buds (Table 1). Most of the sugars in the aborting flower buds showed significant declines in content. Sucrose (Suc), glucose (Glu), and D-fructose (Fru) were the three most abundant sugars in the flower buds, and decreased by 25.6%, 86.5%, and 76.0%, respectively. D-galactose (Gal), inositol (Inositol), and D-arabinose (Ara) were the second most abundant sugars in lotus flower buds, and decreased by 84.3%, 66.3%, and 53.6%, respectively. In addition, some trace sugars including trehalose (Tre), D-sorbitol (Sorbitol), and xylitol (Xylitol) also showed a significant decline in the aborting flower buds. Lactose (Lac) was not detected in the aborting flower buds. Three sugars including maltose (Mal), L-fucose (Fuc), and L-rhamnose (Rha) were increased in the aborting flower buds, by 18.9%, 47.6%, and 71.3%, respectively.

### 2.6. Genome-Wide Identification and Expression Analyses of Lotus miR156 Family Genes

miR156 was a potential regulatory hub for integrating the signals of sugar and flowering control. In a genome-wide approach, we identified 9 precursors of lotus miR156 family members (Table 2). Their optimal secondary structure is shown in Figure S3 with the smallest free energy from −0.64 to −0.43 kcal/mole/nucleotide.

**Figure 5:**
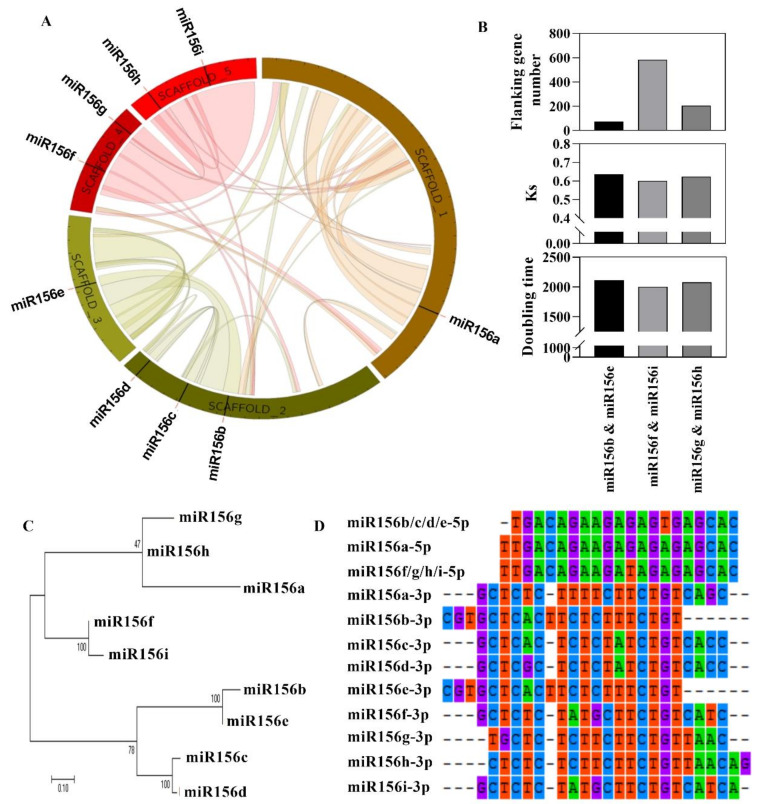
Evolutionary analyses of the lotus miR156 gene family. (**A**) Chromosome location and collinearity of the miR156 gene. (**B**) Duplication event of a large segment of the chromosome. (**C**) Phylogenetic tree of the miR156 gene family. (**D**) Mature sequence of miR156s.

Nine lotus miR156 members were found distributed over five scaffolds (Figure 5A). They did not show a clumped distribution pattern, indicating that members in the lotus miR156 family were not tandemly duplicated genes. We examined the sequence conservation and their surrounding flanking regions among the miR156 family members and three pairs of genes were identified as arising from segmental duplication events, which might have occurred as recently as 2000 years ago (Figure 5B). This is close to the time of the recent whole-genome doubling event in lotus. The evolutionary tree of the lotus miR156 gene family was constructed using the maximum likelihood method (Figure 5C). Most nodes have bootstrap values greater than 70%. We can find that the duplicated gene pairs including miR156b-miR156e, miR156f-miR156i, and miR156g-miR156h are located at same terminal branches of the evolutionary tree, respectively. The expansion of the lotus miR156 gene family was a late event in the evolution of the family. In addition, miR156c-miR156d also appeared in the same terminal branch. They might be the product of another gene expansion event such as transposed duplication. Sequences of miR156-5p could be divided into three classes, including miR156a, miR156b/c, and miR156f/g/h/I, based on sequence similarity (Figure 5D). The miR156-5p sequences of each class were almost the same, but there were large sequence differences in miR156-3p. 

The cis-elements in the promoters of the miR156 family genes were analyzed by PlantCARE (http://bioinformatics.psb.ugent.be/webtools/plantcare/html/, accessed on 18 February 2020) (Table S7). The promoter of miR156 contained a few cis-elements involved in light responses and plant hormone signaling, which play significant roles in regulating the growth and stress responses of plants. Light-responsive elements were the most abundant elements in the promoter of different miR156s, indicating that they were potentially influenced by light.

**Figure 6:**
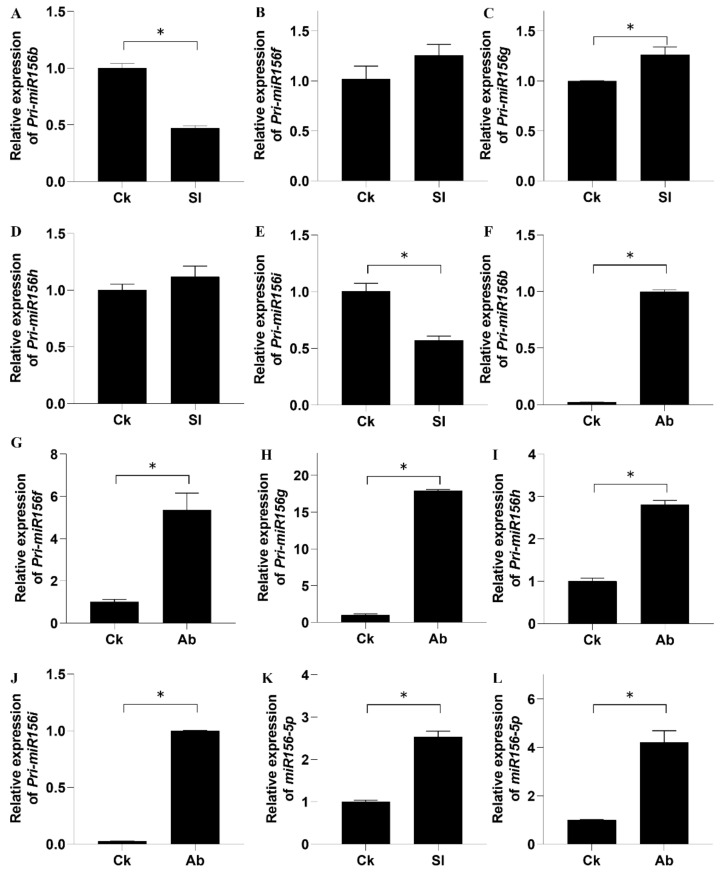
Relative expression levels of miR156s in lotus. The lotus were grown at full light (Ck) and 70% shade (Sl) treatment for 1 month and then leaves were sampled for RT-qPCR analysis of the miR156 family members’ precursors (**A**–**E**) and miR156-5p (**K**). Normal (Ck) and aborting (Ab) flower buds were collected from 1-month-old lotus for RT-qPCR analysis of the miR156 family members’ precursors (**F**–**J**) and miR156-5p (**L**). Values are the means ± SE of three independent experiments with at least three replicates for each. U6 small nuclear RNA was used as an endogenous control to normalize expression levels of miRNAs from RT-qPCR. Asterisks indicate that mean values are significantly different in comparison with Ck at *p* < 0.05 according to a *t*-test.

The expression levels of the miR156 family members’ precursors were analyzed in leaves of shaded lotus, as well as in the normal and aborting flower buds. Results showed that the expression of Pri-miR156f, Pri-miR156g, and Pri-miR156h was increased, while that of Pri-miR156b and Pri-miR156i decreased in the shaded leaves (Figure 6A–E). Pri-miR156a, Pri-miR156c, Pri-miR156d, and Pri-miR156e were not detected, which is consistent with our sequencing results. Compared with the normal flower buds, the precursors of these five members of the 156 family were all increased in the aborting flower buds (Figure 6F–J). The difference in the expression patterns of miR156 family members between leaves and flower buds might be due to tissue specificity.

The expression level of mature miR156-5p was higher in the shaded leaves than in the full-light leaves (Figure 6K). Moreover, in the aborting flower buds, it was higher than in the normal flower buds (Figure 6L), which is consistent with the increased expression levels of miR156 family member precursors in the aborting flower bud.

Taken together, it can be speculated that the miR156 family, especially miR156-5p, was closely related to lotus bud abortion and might participate in the growth and development of flower buds.

### 2.7. In Vivo Gene Function Studies of miR156 and Trehalose-6-P Synthase 1 (TPS1)

**Figure 7:**
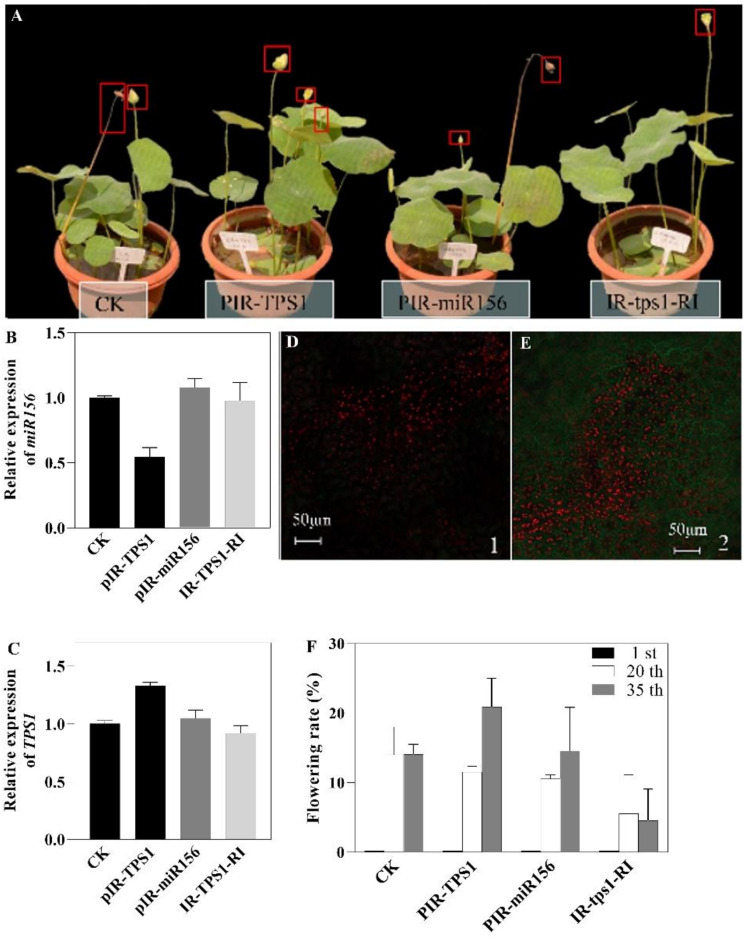
Phenotypic characterization and gene expression analysis of transgenic lotus. (**A**) Lotus transformed for 32 days; (**B**) Relative expression level of miR156; (**C**) Relative expression level of *TPS1*; (**D**,**E**) GFP signal in lotus; (**F**) Flowering rate in 1, 20, and 35 days after transformation.

T6P functions as an indicator of plant carbohydrate status and can regulate the level of miR156. TPS1 is the only TPS enzyme that catalyzes the T6P synthesis reaction and is shown to be required for embryogenesis, vegetative growth, and flowering. Consistent with this, the expression of TPS1 was down-regulated, while the level of miR156 was up-regulated in aborting flower buds with decreasing Tre content. To further elucidate the function of miR156 and TPS1 in lotus flower bud abortion, we transiently modified gene expression through either over-expression (OE) or RNA-interference-mediated down-regulation (RI) based on the IL60 system (Figure 7A). All the transformed plants showed increased expression of transgenes in OE plants and displayed reduced expression levels in RI plants in comparison with the control plants (Figure 7B,C). In addition, green fluorescent protein (GFP) fluorescence could be observed inside veins and mesophyll cells of GFP OE lotus (Figure 7D,E). These results suggested that all transgenes had been successfully overexpressed or silenced in the transiently transformed plants, and therefore could be used for subsequent experiments. Transiently transformed plants showed spatial and temporal variation in morphological characters. TPS1-OE lotus displayed an increased flower:leaf ratio, indicating that fewer flower buds were aborted (Figure 7F). In contrast, TPS1-RI plants had worse flower performance. TPS1-OE lotus had a higher flower:leaf ratio on day 35 after transformation than day 20, which was likely due to the replication and transfer of the IL60-1 plasmid taking an extended period. The flower:leaf ratio significantly decreased in miR156-OE plants on day 20 of transformation. However, there was no significant difference on day 35 of transformation, which might be because miRNA is easily decomposed and the target gene product can gradually accumulate (Figure 7F).

Moreover, the expression level of miR156 in TPS1-OE lotus was down-regulated, indicating that TPS1 might have a regulatory effect on miR156 in lotus (Figure 7B). In addition, the results show that TPS1-RI has little effect on the expression level of miR156. We speculate this could be because TPS1 did not regulate all members of the miR156 family.

## 3. Discussion

### 3.1. Trehalose and Sucrose Metabolism Was Part of the Chain of Events Surrounding Lotus Flower Bud Abortion

Suc and its hydrolysates not only provide energy for the orderly progress of cellular processes, but also serve as starting molecules to be converted into various metabolites and structural units for the synthesis of necessary polymers, including starch, cellulose, callose, and proteins [26]. Their reduction also has a great impact on other sugar. For example, inositol derived from Glu is significantly reduced, as mentioned above, and is involved in important oxidative metabolism pathways in plants, and plant cell wall biosynthesis is inseparable from it [27]. In addition, Ara is a component of cell wall polysaccharides [28], and Gal is one of the components of hemicellulose. The reduction in these sugars leads to the destruction of cell wall integrity, thereby affecting cell division, limiting cell proliferation, and negatively affecting plant growth and development. In addition, soluble sugars can also participate in the osmotic regulation of crops under stress [29]. 

It was remarkable that Tre decreased the most in the aborting flower buds, up to 89.0%, among the detected sugars (Table 1). Tre and its precursor T6P have diverse and vital functions in plants, linking growth and development to carbon status [30,31]. As an important enzyme in T6P synthesis, TPS1 is also essential for normal vegetative growth and transition to flowering, and the deletion of TPS1 prevents floral transition in Arabidopsis [32,33]. They were reported to play important roles in maize early kernel development and events leading to stress-induced kernel abortion [34]. Reduction in TPS1 results in an early increase in miR156 levels [8], which is consistent with our sequencing results. In the aborting lotus flower buds, the expression of TPS1 was down-regulated, while the level of miR156 was up-regulated (Tables S3 and S4). TPS1/T6P might play important roles in mediating the perception of miR156 to sucrose levels or the level of sucrose hydrolysate, and thus miR156 can regulate its target genes timely under low-light stress [35]. In summary, the reduction in carbohydrates (especially Suc and Tre) might be an important cause for flower bud abortion, which ultimately leads to starvation and destroys sugar signals. While there is considerable information about the signaling role of sugars in developmental transitions, the interplay between sugar signaling and other regulatory elements remains poorly understood.

### 3.2. Sucrose Metabolism and Signaling Regulated Flowering

Acid invertase located in cell wall and vacuole can hydrolyze Suc into Glu and Flu and releases hexoses, and is also called beta-fructofuranosidase [36]. As a target gene of miR399a_3, the *β-fructofuranosidase* gene (XM_010260719.2) was significantly down-regulated in the aborting flower buds with the increased expression level of miR399a_3 (Tables S3–S5). Recently, miR399 has been shown to be involved in the regulation of *alkaline/neutral invertase* (*NINV*) genes in *Salvia* *Miltiorrhiza* [37]. Meanwhile, as the inhibitor of acid invertase, *cell wall/vacuolar inhibitor of fructosidase 2-like* was also down-regulated in the aborting flower buds and targeted by novel_mir13 (Tables S3–S5). Beyond metabolism, invertase also plays a signaling role in development. In tobacco, antisense suppression of cell wall invertase (CWIN) expression resulted in pollen sterility [38]. Boyer and McLaughlin [39] also showed that the participation of genes encoding a cell wall invertase and a soluble invertase leads to flower abortion. Additionally, vacuole invertase was also considered to be a key player in cell expansion through osmotic regulation [40]. 

As another key enzyme in sucrose metabolism, Sus reversibly degrades sucrose into uridine diphosphate-glucose (UDPG) and Fru [41]. With UDPG as substrate, UDP-glucose dehydrogenase is the first step of a branched pathway leading to plant cell wall polysaccharides [42]. In this work, *UDP-glucose 6-dehydrogenase 5-like* (XM_010261018.2) was down-regulated and found to be a target of miR156f-5p and miR156a-5p (Tables S4 and S5, Figure 3 and Figure 4). In addition, UDP-galactose is also a derivative of UDPG. Galactinol synthase encodes a key enzyme catalyzing the first committed step in the biosynthesis of raffinose family oligosaccharides (RFOs), being responsible for galactinol formation using UDP-galactose and myo-inositol as substrates [43]. Here, the *galactinol synthase 2-like* gene was significantly down-regulated as its regulator miR6300 expression increased (Table S6, Figure 3 and Figure 4). This might decrease the production of galactinol and RFOs, which are reported to play a role in carbon storage and osmotic adjustments as well as in membrane and protein stabilization [43,44,45].

### 3.3. Loss of Cell Integrity Might Lead to the Flower Bud Abortion

The appearance and inner morphological structure of the aborting lotus flower buds showed that most buds were shriveled [11]. The plant cell wall is a dynamic and highly controlled structure that is essential for growth and development [46]. Lignin is a major component in secondary cell walls, and its reduction affects the integrity of plant cell walls and the strength of stems [47]. It has been reported that laccase (LAC4), AthLAC11, and AthLAC17 contribute to the constitutive lignification of the floral stems in Arabidopsis [48,49]. In our predicted target genes of miR397a_3, three laccase genes, laccase-4-like/-11-like/-17-like were down-regulated (Table S6, Figure 3 and Figure 4). Basic *blue protein-like* and *blue copper protein-like* genes, which also affect the accumulation of lignin [50], were down-regulated in the aborting flower buds and might be targeted by miR408-3p_2 and miR408d (Table S3–S5). 4-coumarate--CoA ligase 2 (4CL2)-like can alter lignocellulose composition without affecting stem growth in Arabidopsis [51]. *4CL2-like* was up-regulated in the aborting flower buds, with a decrease in *miR319a-5p_1* expression (Table S6, Figure 3 and Figure 4). 

Hemicellulose is usually combined with lignin to enhance cell wall strength and is composed of xyloglucan, xylan, and mananns [52]. β-D-xylosidase, one of the xylan-degrading enzymes [53], was significantly up-regulated in the aborting flower buds, while *miR319a-5p_1* was significantly down-regulated (Table S6, Figure 3 and Figure 4). In addition, the transcription factor *bHLH62-like* gene (XM_010244862.2), a target gene of miR166d-5p_2, was correspondingly up-regulated (Table S6, Figure 3 and Figure 4) and is associated with the regulation of polysaccharidic secondary cell wall biosynthesis and cell-autonomous lignification in Arabidopsis [54]. In terms of cell wall degradation, it is brought about by the action of several hydrolytic enzymes. One of these is thought to be polygalacturonase [55]. Here, in lotus aborting flower buds, one target gene of miR164d, the *polygalacturonase-like* gene (XM_010244147.2), was correspondingly up-regulated and is involved in pentose and glucuronate interconversions associated with cell wall degradation (Table S3–S5). 

Membrane integrity could also influence plant growth and development. Several lines of evidence show that membrane integrity is often affected by environmental stress that leads to premature termination of the development of embryos and flowers [56]. In the aborting flower buds, fifteen target genes encoding intrinsic or integral membrane proteins were obviously changed. Among them, aquaporin *TIP2-1* was significantly down-regulated in the aborting flower buds with the up-regulated *miR156_2* levels (Table S6, Figure 3 and Figure 4). As a multifunctional organelle in plant cells, the vacuole plays an important role in the maintenance of the intracellular space and its membrane is highly permeable to water due to its content of aquaporin TIPs, allowing rapid water influx with low activation energy [57]. The osmotic stress caused by the decrease in the aquaporin protein results in a water deficit which serves as a signal to regulate the expression of other genes. Late embryogenesis abundant (Lea) proteins are highly hydrophilic proteins [58]. Lea5 was up-regulated in the abortion flower buds as expression of *miR159b-5p* declined (Table S6, Figure 3 and Figure 4). It was reported that Lea5 was highly induced under drought stress [59], indicating that osmotic stress might induce the abortion of lotus flower buds. Additionally, as the expression of miR6300 increased, its target gene *nonrace-specific disease resistance* 1 (*NDR1*, XM_010269586.2) was down-regulated in the aborting flower buds (Tables S3–S5). NDR1 is a plasma-membrane-localized protein and is confirmed to be involved in preventing fluid loss and maintaining cell integrity through plasma membrane–cell wall adhesions in Arabidopsis [60].

Three target genes of miR156_2, including *probable receptor-like protein kinase At2g42960* (XM_010245405.2), *transmembrane protein 18* (XM_010258025.1), and *cationic amino acid transporter 1-like* (*CAT1*, XM_010278577.2), were all down-regulated and are associated with membrane function and participate in carbohydrate transport and partitioning (Tables S3–S5). As an amino acid transporter, CAT1 was reported to be expressed in seeds and vascular tissue, such as peduncle, for example [61], as amino acids are distributed through the xylem and phloem to supply sink organs [62]. Probable receptor-like protein kinase (At2g42960) was predicted to play roles in protein kinase activity and binding. Transmembrane protein 18 is associated with the transport of specific substances across the membrane. It seems that nutrient transport and partitioning in the aborting flower buds were reduced.

### 3.4. Cell Proliferation and Cell Expansion Were Influenced by Shading

Cell proliferation and expansion are crucial for flower bud growth and development. With the up-regulated expression of *miR156_2*, *miR169r-5p*, *miR156_2*, and *miR6300*, four cell-cycle-related target genes including *serine/threonine-protein kinase Aurora-3*, putative deoxyribonuclease *TATDN1*, *cyclin-D4-1-like*, and putative *cyclin-D6-1-like* were down-regulated, respectively (Tables S3–S5). Aurora kinases regulate cell proliferation by controlling M-phase events [63]. It is reported that aurora was able to drive continuous flowering in plants [64]. TATDN1 plays an important role in chromosomal segregation and cell cycle progression [65]. Moreover, cyclin-D4-1-like and putative cyclin-D6-1-like, belonging to the D-type cyclins that can perceive the cell’s environment, act as growth sensors to promote initiation and/or establishment of the postmitotic interphase (G1) phase [66]. The expression of plant D-type cyclins is responsive to growth factors like hormone and carbohydrate [67]. A Passiflora homolog of a *D-type cyclin* gene is differentially expressed in response to sucrose, auxin, and cytokinin [68].

According to the KEGG pathway analysis, we identified 26 genes involved in plant hormone signal transduction and regulating plant growth and cell death (Table S6, Figure 3 and Figure 4). Nineteen of them were up-regulated in the aborting flower buds and were targeted by miR156. Seven genes were targeted by miR167d_1 and identified as down-regulated (Table S6). Among them, the SPL family genes and *TGA1* gene can repress cell elongation and cell division by inhibiting the activity of Brassinosteroid Insensitive 1 (BRI1). As the target genes of miR167d_1, ARF6 and ARF8 are auxin-response factors (ARFs) [69]. A recent study confirmed that down-regulation of *ARF6* and *ARF8* by miR167 leads to floral development defects and female sterility in tomatoes, which suggested that they have conserved roles in controlling the growth and development of vegetative and flower organs in dicots [70]. Moreover, one target gene of miR396a-5p, IAA-amino acid hydrolase *ILR1-like 6*, was down-regulated (Table S6, Figure 3 and Figure 4), which might result in lower free auxin levels in lotus flower buds. The decreased levels of free auxin might cause the down-regulation of *miR167*, which then releases the repression of its targets, ARF6 and ARF8 [71].

The development of flower buds stops with the cessation of cell expansion. Ethylene also plays a key role in cell expansion in plant developmenti. Here, we identified an ethylene-responsive transcription factor gene *ERF020* that was up-regulated in the aborting flower buds (Table S6, Figure 3 and Figure 4) as its regulator *miR319a-5p_1* was down-regulated.

Several studies have shown that down-regulating the expression level of *miR164* ethylene releases the repression of NAC domain-containing proteins, which then perform roles in suppressing cell expansion [72]. Here, *miR164* was significantly down-regulated in the aborting flower buds while three NAC domain transcription factor target genes (LOC10459793, LOC104598088, and LOC104598350) were up-regulated.

In addition, miR169 and its target gene transcription factor *nuclear transcription factor Y subunit A* (*NF-YA*) play an important role in plant growth. It is reported that overexpression of Arabidopsis *NF-YA4* leads to slower growth and a delayed flowering phenotype [73]. Here, *NF-YA10-like* (LOC104601425) was also up-regulated and positively correlated with *miR169r-5p* (Tables S3–S5). Previous studies have shown that the miR169d/NF-YA2 (10) module plays an important role in Arabidopsis thaliana early flowering induced by stress [74].

### 3.5. DNA Repair, RNA Editing, and Nucleocytoplasmic Transport Events Steeply Reduced in the Aborting Flower Buds

In the aborting flower buds, some genes that affected DNA repair, RNA editing, and nucleocytoplasmic transport in lotus changed significantly, which could be the reason for the acceleration of programmed cell death (PCD) in abortive flower buds (Figure 2). Similar gene expression changes were also reported in the crown tissues of the type II necrosis line under low-temperature conditions, where a number of cell-division- and DNA-repair-related genes were specifically down-regulated [75]. The target gene *DNA damage repair/tolerance protein* *100* (*DRT100*) of miR397 belongs to the plant DRT100 protein family [76] and was significantly down-regulated in aborting flower buds. It is reported that grape DRT100 protein may play an important role in DNA damage repair and tolerance induced by UV-B [76]. 

Pentapeptide repeat (PPR) proteins are a large family of RNA-binding proteins that are very important for the expression of the organelle genome and organelle biogenesis [77]. Here, four PPR genes, including *PPRDOT4* (LOC104586410), *PPRAt1g56570* (LOC104607063), *PPRat2g04860* (LOC104609601), and *PPRAt1g69350* (LOC104598024), were significantly down-regulated, and the expression levels of their regulator miRNAs, *miR156_2*, *miR157d-3p*, and *miR6300*, increased, respectively (Tables S3–S5). Among them, PPRDOT4 mediated chloroplast RNA editing [78]. It has been reported that RF-PPR592, which belongs to the PPR protein family, can restore the fertility of cytoplasmic male sterile plants [79]. To cope with stress, the cells underwent drastic changes and the exchange of cellular information intensified. 

The exchange of macromolecules inside and outside the nucleus is mediated by nuclear pore complexes (NPC) embedded in the nuclear membrane [80]. Down-regulation of miR395b_3 increases the expression level of nuclear pore complex protein NUP133. NUP133 is a component of NCP and encodes nuclear pore protein (Nucleopin, NUP) [81]. In addition to the role of NPCs and NUP in nucleoplasmic transport, they are also involved in many physiological processes, including kinetochore and spindle assembly, cell cycle control, gene expression regulation, chromatin tissue, DNA repair, and DNA replication [82,83].

### 3.6. A Potential Regulatory Network of Lotus Flower Bud Abortion Centered on miR156

**Figure 8:**
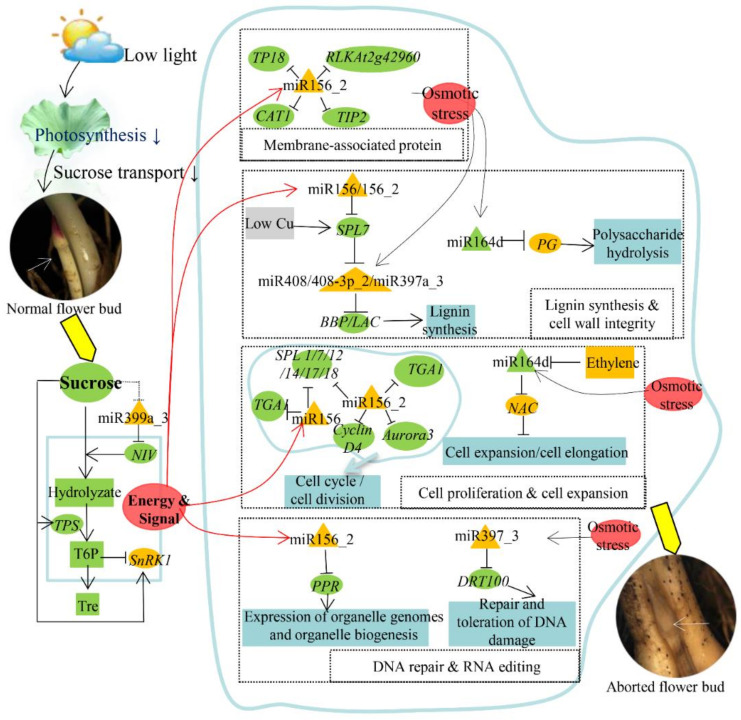
miR156-mediated potential mechanism of lotus flower bud abortion under low light. Under low-light conditions, the photosynthesis of lotus plants is weakened, and the sucrose transported from leaves to flower buds is reduced, eventually causing flower buds to die due to starvation. During the abortion process of flower buds, a series of miRNAs are involved. Among them, miR156 family plays central role—by sensing sugar signals, it regulates the target genes and participates in many aspects related to the growth and development of flower buds together with other miRNAs (such as miR408, miR397, and miR164) and their targets, including sugar metabolism, cell wall integrity, the activity of biomembranes, cell proliferation/expansion, and so on. Abbreviation: *NIV*, Beta-fructofuranosidase, soluble isoenzyme I-like; *TPS*, Trehalose-6-P synthase; *SnRK1*: Sucrose non-fermenting-1-related protein kinase 1; *Tre*, Trehalose; *TP18*, Transmembrane protein 18; *CAT1*, cationic amino acid transporter 1-like; *RLKAt2g42960*, probable receptor-like protein kinase At2g42960; *TIP2*: Aquaporin Tonoplast Intrinsic Protein2-1; *BBP*, Basic blue protein-like gene; *LAC*, Laccase gene; *PG*, Polygalacturonase-like gene; *SPL*, Squamosa promoter-binding-like protein; *TGA1*, Teosinte glume architecture 1; *Cyclin* *D4*, Cyclin-D4-1-like; *Aurora3*, Aurora-3 gene; *NAC*, NAC domain transcription factor target genes; *PPR*, Pentapeptide repeat proteins; *DRT100*, DNA-damage-repair/toleration protein DRT100-like; Low Cu, Low Copper Signal.

Low-light intensity appears to have a major effect in inducing lotus flower bud abortion. In this work, we showed that ten carbohydrates in the aborting flower buds were reduced, with Tre/T6P decreased the most (Table 1). Tre/T6P was demonstrated to be at the center of the sucrose homeostatic mechanism that determined the allocation and use of sucrose and other carbohydrates. As sugars are both metabolites and signaling molecules, carbon starvation might have induced a series of miRNA and target gene changes which regulated many aspects of flower development and eventually lead to flower bud abortion. Among them, the miR156 family played an important role in the response of sugar signaling [84]. In this work, our results demonstrated that miR156 is located in the center of the regulatory network of lotus flower bud abortion (Figure 8). 

In the apical meristem, the T6P pathway can inhibit the expression of *miR156* [22]. MiR156 might participate in lignin synthesis by down-regulating *SPL7*, which is also under control of other miRNAs, such as miR397 and miR408 (Tables S3–S5, Figure 3 and Figure 4). In addition to lignin, xylose, polysaccharides, and glycoproteins responsible for cell wall integrity were also influenced by the expression changes of *miR319a-5p1*, *miR164d*, *miR172e-3p*, and their related targets (Tables S3–S5, Figure 3 and Figure 4). Moreover, invertase-gene-encoding acid invertase was significantly down-regulated by miR399a_3 (Table S3–S5, Figure 3 and Figure 4). Previous studies have shown that down-regulation of cell wall invertase and soluble invertase depletes the ovarian sugar pool, resulting in up-regulation of the *ribosome inactivating protein* and *phospholipase* genes [39]. The latter change seems to initiate senescence and the degradation of the cell membrane, leading to irreversible abortion [39].

The integrity of the cell wall is inseparable from the integrity of plasma membrane. MiR6300 targets the *NDR1* gene (Tables S3–S5, Figure 3 and Figure 4), which plays an important role in plasma membrane–cell wall adhesion by preventing fluid loss and maintaining cell integrity [60]. Amongst the predicted mRNA targets of miR6300, the *NDR1* gene plays a significant role in preventing fluid loss and maintaining cellular integrity [60]. Another membrane-related protein [57], *aquaporin*, was also decreased under the regulation of miR156_2, which might result in water loss together with miR6300 and its targets (Tables S3–S5, Figure 3, Figure 4 and Figure 8).

In general, some adaptive cellular responses to specific stress conditions are interrelated with other environmental responses [85]. Many miRNAs respond to osmotic stress or water deficit, such as miR159, miR164, miR167, miR398, and miR408 [86]. The decrease in lignin might affect the function of vascular tissue and the function of sucrose transport of phloem.

Moreover, the increase in *miR156*/*miR156_2*, *miR167*, *miR169r-5p*, *miR396a-5p* and *miR6300* in the aborting flower buds might down-regulate an extensive array of cell-cycle-control-related target genes and thus inhibit the growth of flower buds (Figure 8). In addition, cell expansion is also important for flower bud growth and development. Our results show that miR164d, miR169e_3/169h_2, miR169r-5p, and miR172e-3p might be involved in the regulation of cell expansion (Tables S3–S5, Figure 3 and Figure 4).

In the process of flower bud abortion, some stress-responsive miRNAs and their target genes were also up-regulated, such as *miR159b-5p* and its target gene *Lea5* and *miR168*/*168a-5p* and *Lr10* (Tables S3–S5, Figure 3 and Figure 4). Although these resistance genes were expressed under stress, they could not only reverse the situation of abortion, but also increase the consumption of sugar, which might accelerate the abortion of flower buds. On the other hand, the up-regulation of these genes was also a positive response of plants to shade stress and reduced the reproductive process with low success. It is worth noting that some miRNAs and genes with only trace expression levels might also play important roles. A new expression analysis technique might have become available to monitor these genes [87].

To summarize, these results demonstrated that low-light-intensity-induced flower bud abortion might be explained by dysregulated-sugar-metabolism-triggered degradation of the cell wall and cell membranes, which ultimately causes irreversible abortion of flower buds (Figure 8). A miR156-centric regulatory network containing several miRNAs and corresponding target genes play major roles in regulating the process and control multiple aspects of biological processes including sugar metabolism, cell wall integrity, the activity of biomembranes, cell proliferation, and so on. The relationship between miR156 and sugar signaling awaits further studies. IL-60-gene-expression-system-mediated TPS1 overexpression showed great potential in the practical application of improving lotus flowering performance. Overall, our study identified the genetic basis for how lotus produces so many aborted flower buds, facilitating genetic improvement of the shade tolerance of lotus.

## 4. Materials and Methods

### 4.1. Plant Materials 

The lotus “Boli Furen” used in this study was planted in a large pool of 2 m × 2 m (32° N 119° E, Nanjing, China) under natural light conditions in April, and was sampled in June when there were enough leaves to obtain flower buds. Flower buds with similar morphological size and at the same differentiation stage were collected based on the method of sampling and identification of the flower bud differentiation period in vivo invented by our laboratory [11]. Three biological replicates were collected and each independent repeat contained multiple flower buds, about 0.5 g. The samples were snap frozen in liquid nitrogen and stored in the refrigerator at −80 °C for subsequent small RNA sequencing and quantitative detection of sugar. Lotus “Xue Lian14” was used in the transient transformation experiment and was planted in 38 cm-diameter pots at the end of April. After the plants grow about 3 standing leaves, they were shaded. The shaded scaffold was 2 m high, three-pin shading nets were laid on the roof and surroundings, and the shading rate was about 70%.

### 4.2. RNA-Seq Library Construction, Sequencing, and Analysis

For RNA-seq, the normal (Ck) and aborting lotus flower buds (Ab) (2–3 cm in length) were collected with three biological replicates. Total RNA was isolated using the Cetyltrimethyl Ammonium Bromide (CTAB) method [88]. Six small RNA libraries and six mRNA libraries were separately constructed and sequenced by BGI Tech Solutions according to the standard procedure. In order to obtain reliable sequencing data, we carried out strict quality control at each step of the experiment. The experiment pipeline is shown in Figure S4. Lotus genome (https://www.ncbi.nlm.nih.gov/genome/?term=Nelumbo+nucifera, accessed on 1 August 2019) and sRNA databases were used for reads mapping. To make sure every unique small RNA was mapped to only one category, we followed this priority rule: MiRbase > pirnabank > snoRNA(plant) > Rfam > other SRNA. miRA was used to predict novel miRNA by exploring the characteristic hairpin structure of miRNA precursor [89]. Expression values of gene transcripts are represented as FPKM (fragments per kilobase of exon per million fragments mapped). The expression levels of both known miRNA and novel miRNA from each sample were calculated and normalized to transcripts per million (TPM). The statistical significance of miRNA expression changes were estimated using the DEGseq R package v1.18.0 [90]. Genes were considered significantly up- or down-regulated over the control when the adjusted *p*-value (Q-value) < 0.001 and log_2_ fold changes ≥2. Sequence data have been deposited with the GenBank data libraries under accession number PRJNA855162 and PRJNA707244 [11].

### 4.3. miRNA Expression Profiles and Their Target mRNAs

In order to find more accurate targets, we used psRobot [91] and TargetFinder [92] to predict miRNA targets. In addition, based on two omics, small RNA data and transcriptome data, the association between miRNA and its target genes was further analyzed, where the transcriptome was sequenced simultaneously using the same samples as the small RNA sequencing. Generally, miRNAs were negatively correlated with target genes; therefore, the correlation coefficient calculated by the R packages [93] between miRNAs and target genes was negative, and the multiple of the difference between miRNA and its target gene was one positive and one negative respectively in the same difference group. Based on the above, the differentially expressed miRNAs and differentially expressed target genes most relevant to flower bud abortion were screened.

### 4.4. Functional Enrichment Analysis of Target mRNAs

GO enrichment analysis and KEGG pathway analysis were performed to the target mRNAs of differentially expressed miRNA (DEM) to comprehensively determine their biological functions. All DEM target genes were mapped to GO terms in the Gene Ontology database (http://www.geneontology.org/, accessed on 1 August 2019), then the enriched significant GO terms (taking a correct *p*-value ≤ 0.05 as a threshold) compared to the lotus genome background were categorized into three levels, “biological process”, “cellular component” and “molecular function” [94]. Pathway-based analysis helped to further understand their biological functions. KEGG was used to perform pathway enrichment analysis [95]. This analysis identified significantly enriched metabolic pathways or signal transduction pathways in the DEM target genes when compared with the whole-genome background. The calculating formula was the same as that in GO analysis, and the KEGG pathway were defined as significant when satisfying a corrected *p*-value ≤ 0.05.

### 4.5. Quantitative PCR for miRNAs and mRNAs

The expression levels of mature miRNAs and mRNA involved here were validated by quantitative PCR. Synthesis of the first strand of cDNA of miRNA was performed with a Mir-X miRNA First-Strand Synthesis Kit (Code No. 638315, TaKaRa, Tokyo, Japan) with U6 (Small nuclear RNA, snRNA) serving as an internal control. The forward primer for RT-qPCR validation of miRNAs was designed to match the candidate miRNAs, and the reverse primers were as provided by the kit. The first strand cDNA of the mRNA was synthesized by using the PrimeScript TM RT reagent Kit with the gDNA Eraser (Perfect Real Time) (Code No. RR047A, Takara, Tokyo, Japan), with the *actin* gene (XM_010252745) for normalization. The primers of the miRNAs, mRNAs and internal control genes are listed in Supplementary Table S8. The RT-qPCR reactions were performed using a Mastercycler ep realplex real-time PCR system (Eppendorf, Hamburg, Germany) with TB Green^®^ Premix Ex Taq TM II (Tli RNaseH Plus) (Code No. RR820Q, TaKaRa, Tokyo, Japan) according to the manufacturer’s instructions. CT values obtained through quantitative PCR were analyzed using 2^−ΔΔCT^ methods to calculate the relative fold-change values [96].

### 4.6. Determination of Sugar in Flower Buds

The normal and aborting flower buds were also used for sugar analysis with at least three biological replicates. The freeze-dried flower buds were crushed using a mixer mill (MM400, Retsch, Haan, Germany) with a zirconia bead for 1.5 min at 30 Hz. Then, 20 mg of powder was diluted to 500 μL methanol: isopropanol: water (3:3:3:2 *v*/*v*/*v*), vortexed for 3 min, and ultra-sonicated for 30 min. The extract was centrifuged at 14,000 rpm at 4 °C for 3 min. Then, 50 μL of the supernatants were collected and were evaporated under nitrogen gas stream and transferred to the lyophilizer for freeze-drying. The residue was used for the further derivatization. 

The derivatization method was as follows: the sample of small molecular carbohydrates was mixed with 100 μL solution of methoxyamine hydrochloride in pyridine (15 mg/mL). The mixture was incubated at 37 °C for 2 h. Then, 100 μL of bis(trimethyl-silyl) trifluoroacetamide (BSTFA) was added into the mixture and kept at 37 °C for 30 min after vortex-mixing. The mixture was diluted to the appropriate concentration with n-hexane. The mixture was stored in brown injection bottles for gas chromatography mass spectrometry (GC-MS) analysis.

### 4.7. miR156 Precursor Prediction, Cloning, and Cis-Element Analysis in the Promoter Region

The mature and precursor sequences of the miR156 family members were obtained from the lotus miRNA and transcriptome databases held in our laboratory. The UNAFold v3.8 suite (http://unafold.rna.albany.edu/, accessed on 18 February 2020) was used to predict the secondary structure of the miR156 precursor. In addition, different libraries were compared to verify the accuracy of the miRNA matures. Generally, the 2 kb region upstream of the gene is the promoter region and contains many cis-acting elements. The 2 kb sequence upstream of each of the miR156 gene family was found and then analyzed using PlantCARE (http://bioinformatics.psb.ugent.be/webtools/plantcare/html/ accessed on 1 August 2019), respectively.

The CTAB method was used to extract DNA from fresh leaves of lotus. The primers of the miR156 family precursor are listed in table X and the products were connected to the pEasy Blunt carrier (Code No. CB101, TransGen, Beijing, China) for use.

### 4.8. Reconstruction and Transformation of Related Overexpression/Interference Vectors

The IL60 system derived from tomato yellow leaf curl virus (TYLCV) is an effective expressing/silencing system that can spread from the infected site to the entire plant and be expressed stably for a long time, but cannot be inherited by plant offspring and has the characteristics of safety, broad spectrum, and high efficiency [97]. With IL-60-1 as the disarmed helper ‘‘virus’’, PIR-X replicates, transfers, and is expressed in plants. In addition, inserting the bi-directional promoter “IR” of PIR-X into the other side of the target gene reversely constitutes an interference vector called “IR-X-RI”. The construction of vectors adopts the method of homologous recombination with the TreliefTM SoSoo Cloning Kit Ver.2 (Code No. TSV-S2, TsingKe, Bejing). GFP was cloned from pFast-R05. The fragment of 255 bp (815-1069 bp) selected from the conserved domain of TPS1 (GenBank accession no: XM_010275120.2) was used as the interference fragment, called tps1. miR156g was used to construct PIR-miR156. The table lists the primers for GFP, TPS1, and MiR156g with homology arms, and the *Sal*I cleavage site on the PIR-X vector was selected. While tps1 was PCR-amplified, adding a *Sal*I restriction site to one end and an *Xba*I site to the other, the reverse sequence of “IR” was synthesized and connected to the vector using the *Xba*I restriction site by General Biosystems Company (Chuzhou City, Anhui Province, China).

*E. coli* cells were transformed with the pertinent virus plasmid construct and propagated under ampicillin selection, and the construct was extracted by standard procedures [11]. A micro-syringe of approximately 200 ng DNA (in 100 mL) was used to inject the leaf vein of the recipient lotus plant. The GFP signal in the lotus leaf was observed using a confocal microscope. Lotus root near the top bud was used as a sample to detect the gene expression level of each transformed plant.

## Figures and Tables

**Table 1:** Contents of detected sugars in the normal and aborting flower buds.

Sample	Ck	Ab	Ab/Ck	Q1	Q2
Ara	0.208 ± 0.081	0.096 ± 0.033	0.46	0.437	0.427
Fru	23.566 ± 1.715	5.653 ± 1.690	0.24	7.940	7.600
Fuc	0.025 ± 0.009	0.037 ± 0.008	1.48	0.316	0.313
Gal	3.473 ± 0.362	0.544 ± 0.206	0.16	1.450	1.350
Glu	14.700 ± 1.023	1.980 ± 0.644	0.13	2.520	2.450
Inositol	1.490 ± 0.254	0.502 ± 0.125	0.34	0.838	0.795
Lac	0.025 ± 0.003	N/A	-	0.247	0.253
Mal	0.430 ± 0.046	0.512 ± 0.174	1.19	1.370	1.250
Rha	0.015 ± 0.000	0.026 ± 0.009	1.73	0.331	0.325
Sorbitol	0.005 ± 0.002	0.002 ± 0.001	0.4	0.267	0.268
Suc	18.533 ± 1.811	13.783 ± 4.176	0.74	19.700	17.500
Tre	0.015 ± 0.005	0.001 ± 0.000	0.07	0.187	0.180
Xylitol	0.003 ± 0.001	0.002 ± 0.000	0.67	0.256	0.253

Note: Ck and Ab are normal flower buds and aborting flower buds, respectively; Q1/2 are quality control samples; the unit of sugar content is mg/g; N/A means the sugar was not detected, either because the sugar content in the sample was below the detection limit of the instrument or the sugar was not contained in the sample.

**Table 2:** Information of lotus miR156 family members.

Member	Chromosome	Start	End	Orientation	sRNA Length	Genomic Hits	Hairpin Length
miR156a	megascaffold_1	135192536	135192556	Minus	21	5	88
miR156b	megascaffold_2	57303178	57303197	Minus	20	4	82
miR156c	megascaffold_2	73898371	73898390	Plus	20	4	95
miR156d	megascaffold_2	91786125	91786144	Minus	20	4	95
miR156e	megascaffold_3	31914122	31914141	Plus	20	4	80
miR156f	megascaffold_4	18267161	18267181	Minus	21	4	101
miR156g	megascaffold_4	37030573	37030593	Minus	21	4	99
miR156h	megascaffold_5	7616122	7616142	Plus	21	4	93
miR156i	megascaffold_5	29397707	29397727	Plus	21	4	102

## Data Availability

The data that support the findings of this study are available from the corresponding author upon reasonable request.

## Supplementary Materials

The following supporting information can be downloaded at: https://www.mdpi.com/article/10.3390/ijms23179925/s1.
