# Supplementary material for: Integrated Transcriptome and Targeted Metabolite Analysis Reveal miRNA-mRNA Networks in Low-Light-Induced Lotus Flower Bud Abortion
Source: Int J Mol Sci. 2022 Sep 1;23(17):9925. doi: 10.3390/ijms23179925 (PMC9456346; doi:10.3390/ijms23179925)
Supplement: Supplementary file 1 [file ijms-23-09925-s001.zip › ijms-1845439-supplementary.pdf]

International Journal of *Molecular Sciences*

MDPI

Article

Integrated transcriptome and targeted metabolite analysis reveal miRNA-mRNA networks in low light-induced lotus flower bud abortion

Huihui Ren [1†], Yingchun Xu [1†], Hongsheng Lixie, Jiaying Kuang, Yanjie Wang and Qijiang Jin*

College of Horticulture, Nanjing Agricultural University, Nanjing 210095, China
* Correspondence: jqj@njau.edu.cn (J.Q.J.)
† These authors contributed equally to this study

Supplementary material:

Table S1 Summary of high-throughput sequencing datasets.

Sequencing Type	Sample name	Raw tag count (Mb)	Clean tag count (Mb)	Q20 of clean tag (%)	Percentage of clean tag (%)	Mapped tag (Mb)	Mapped percentage (%)
sRNA	Ab1	2.82	2.51	99	89.12	2.3	92.77
	Ab2	3.00	2.76	99	92	2.52	91.22
	Ab3	2.99	2.67	99.2	89.26	2.41	90.23
	Ck1	2.84	2.55	99.1	89.69	2.30	90.13
	Ck2	2.90	2.62	99	90.28	2.39	91.20
	Ck3	2.94	2.65	99.1	90.06	2.41	91.08
mRNA	Ab1	67.47	62.4	97.78	92.49	57.30	91.83
	Ab2	69.96	65.19	97.7	93.17	60.69	93.09
	Ab3	69.96	64.36	97.62	91.99	59.45	92.37
	Ck1	69.96	64.22	97.66	91.79	60.21	93.75
	Ck2	69.96	64.15	97.68	91.68	59.60	92.90
	Ck3	69.96	63.55	97.7	90.83	59.33	93.36

Table S2 Summary of read mapping of small RNA sequencing.

Type	Abs total count	Percentage	Cks total count	Percentage
total	79428450	100.00%	78138773	100.00%
intergenic	55380749	69.72%	52989557	67.81%
mature	2360895	2.97%	2673100	3.42%
Rfam other sncRNA	27884	0.04%	14218	0.02%
snRNA	21419	0.03%	3928	0.01%
unmap	6777616	8.53%	7128962	9.12%
intron	13009400	16.38%	13233788	16.94%
rRNA	395614	0.50%	421192	0.54%
hairpin	206584	0.26%	244385	0.31%
snoRNA	3484	0.00%	3743	0.00%
precursor	36551	0.05%	33326	0.04%
exon	1204308	1.52%	1388719	1.78%
tRNA	3946	0.00%	3855	0.00%

Table S7 The cis-elements in promoters of miR156 family genes

Motif Annotation	Motif name	*>Nn-miR156a*	*>Nn-miR156b*	*>Nn-miR156c*	*>Nn-miR156d*	*>Nn-miR156e*	*>Nn-miR156f*	*>Nn-miR156h*	*>Nn-miR156i*	count
Basic component	CAATBOX1	○	○	○	○	○	○	○	○	9
	CCAATBOX1	○	○	○	○	○	○	○	○	9
	GATABOX	○	○	○	○	○	○	○	○	9
	POLASIG1	○	○	○	○	○	○	○	○	9
	POLASIG3	○	○	○	○	○	○	○	○	9
	ROOTMOTIFTAPOX1	○	○	○	○	○	○	○	○	9
	EBOXBNnAPA	○	○	○	○	○	○	○	○	9
	MYBCORE	○	○	○	○	○	○	○	○	9
	GT1CONSENSUS	○	○	○	○	○	○	○	○	9
	IBOXCORE	○	○	○	○	○	○	○	○	9
	TATABOX5	○	○	○	○	○	○	○	○	9
	POLLEN1LELAT52	○	○	○	○	○	○	○	○	9
	DOFCOREZM	○	○	○	○	○	○	○	○	9
	RAV1AAT	○	○	○	○	○	○	○	○	9
	GTGANTG10	○	○	○	○	○	○	○	○	9
	TAAAGSTKST1	○	○	○	○	○	○	○	○	9
	WBOXATNPR1	○	○	○	○	○	○	○	○	9
Hormone regulation	MYCCONSENSUSAT	○	○	○	○	○	○	○	○	9
	MYB1AT	○	○	○	○	○	○	○	○	9
	WBOXHVISO1	○	○	○	○	○	○	○	○	9
	WRKY71OS	○	○	○	○	○	○	○	○	9
	CACTFTPPCA1	○	○	○	○	○	○	○	○	9
	GT1GMSCAM4	○	○	○	○	○	○	○	○	9
	ARR1AT	○	○	○	○	○	○	○	○	9
	WBOXNTERF3	○	○	○	○	○	○	○	○	9
	NODCON1GM	○	○	○	○	○	○	○	○	9

	NODCON2GM	○	○	○	○	○	○	○	○	9
	OSE1ROOTNODULE	○	○	○	○	○	○	○	○	9
	OSE2ROOTNODULE	○	○	○	○	○	○	○	○	9
	CPBCSPOR	○	○	○	○	○	○	○	○	9
	SEF4MOTIFGM7S	○	○	○	○	○	○	○	○	8
	MYBST1		○	○	○	○	○	○	○	8
	PYRIMIDINEBOX-OSRAMY1A	○	○		○	○	○	○	○	8
	BOXIINTPATPB	○	○	○	○		○	○	○	8
	REALPHALGLHCB21	○	○	○	○	○		○	○	8
	INRNTPSADB	○	○		○	○	○	○	○	8
	MYB2CONSENSUSAT	○	○	○	○		○	○	○	8
	ACGTATERD1	○	○	○		○	○	○	○	8
	GAREAT	○	○	○	○	○	○		○	8
	CURECORECR	○	○		○	○	○	○	○	8
	SURECOREATSULTR11	○	○	○	○	○		○	○	8
	ASF1MOTIFCAMV	○	○	○		○	○	○		7
	MARTBOX	○	○		○	○	○	○		7
	POLASIG2	○	○	○	○		○		○	7
	TATABOX4	○	○	○	○	○		○		7
	-300ELEMENT		○	○	○	○	○		○	7
	ELRECOREPCRP1		○	○	○	○	○		○	7
	LTRECOREATCOR15		○		○	○	○	○	○	7
	CIACADIANLELHC	○	○			○	○	○	○	7
	RYREPEATBNnAPA		○	○	○	○	○	○	○	7
	ANAERO1CONSENSUS	○	○		○	○	○	○	○	7
	ANAERO2CONSENSUS	○	○	○	○		○	○		7
	EECCRCAH1	○		○	○	○	○		○	7
	BIHD1OS	○	○	○	○		○	○		7
	MYBCOREATCYCB1	○	○		○	○	○	○	○	7
	RHERPATEXPA7	○		○	○	○	○	○	○	7
	SEF3MOTIFGM	○				○	○	○	○	6
	GT1CORE	○	○		○		○		○	6
	CANBNnAPA			○	○	○	○	○	○	6
	S1FBOXSORPS1L21	○	○	○	○				○	6
	NTBBF1ARROLB	○		○		○	○		○	6
	DPBFCOREDCDC3	○	○			○	○		○	6
Stress response	TATAPVTRNALEU	○	○	○	○			○		6
	TBOXATGAPB		○		○	○	○		○	6
	TATCCAOSAMY		○	○			○	○	○	6
	GAGAGMGSA1	○	○		○	○	○	○		6
	DRECRTCOREAT		○		○	○	○	○		6
	GAGA8HVBKN3	○	○		○	○	○	○		6
	CARGCW8GAT	○		○	○	○	○			6
	CTRMCAMV35S	○	○		○	○	○	○		6
	SITEIIATCYTC	○		○		○	○		○	6
	SORLIP2AT	○	○	○		○	○		○	6
	CBFHV		○		○	○	○	○		6
	PRECONSCRHSP70A		○		○	○	○	○	○	6
	TATABOX3	○	○	○				○	○	5
	IBOX	○			○		○	○	○	5

2SSEEDPROTBANAPA			○		○	○	○	○	5
MYBPZM	○			○			○	○	5
ARFAT	○	○		○	○				5
AACACOREOSGLUB1			○		○	○	○		5
SEBFCONSSTPR10A		○		○	○	○			5
-10PEHVPSBD	○		○		○	○			5
ABRELATERD1	○		○		○		○	○	5
CAREOSREP○		○	○		○	○	○		5
IBOXCORENT	○			○		○	○	○	5
SREATMSD				○	○		○	○	5
E2FCONSENSUS	○		○	○	○			○	5
BOXLCOREDCPAL		○		○	○		○		5
AMYBOX1		○			○	○		○	4
RYREPEATLEGUMIN-BOX		○	○				○	○	4
RYREPEATGMGY2		○	○				○	○	4
SV40COREENHAN	○					○		○	4
RBCSCONSENSUS	○		○					○	4
CCA1ATLHCB1		○				○	○		4
MYBPLANT	○	○					○	○	4
MYCATRD22		○	○			○			4
MYBGAHV		○			○	○		○	4
WBBOXPCWRKY1	○	○			○	○			4
TATABOXOSPAL			○	○			○		4
DRE2COREZMRAB17		○		○	○	○			4
MYCATERD1		○	○			○			4
WBOXNTCHN48		○	○		○			○	4
-300CORE						○	○		3
NAPINMOTIFBN		○						○	3
TATABOX2			○					○	3
MYB2AT			○			○			3
QELEMENTZMZM13		○				○		○	3
PROXBBNnAPA			○				○	○	3
TELOBOXATEEF1AA1					○		○	○	3
CATATGGMSAUR				○		○		○	3
CARGATCONSENSUS		○	○			○			3
PREATPRODH	○		○					○	3
UP2ATMSD					○		○	○	3
SORLIP1AT			○	○		○			3
XYLAT	○			○				○	3
INTRONLOWER				○		○			2
ACGTABOX	○		○						2
PALBOXAPC								○	2
LTREATLTI78		○			○				2
MYBATRD22	○							○	2
SP8BFIBSP8BIB	○								2
CGACGOSAMY3							○		2
S1FSORPL21	○							○	2
GCN4OSGLUB1			○					○	2
ACGTOSGLUB1					○			○	2
RAV1BAT	○				○				2
REBETALGLHCB21		○						○	2

	UPRMOTIFIIAT	o							o	2
	GCCCORE		o							2
	LEAFYATAG	o					o			2
	WUSATAg	o							o	2
	MYB1LEPR		o	o						2
	P1BS			o					o	2
	UP1ATMSD	o		o						2
	ANAERO3CONSENSUS			o						2
	SORLIP5AT					o	o			2
	SORLREP3AT			o				o		2
	SEF1MOTIF							o		1
	AMYBOX2						o			1
	CEREGLUBOX2PSLEGA			o						1
	ERELEE4							o		1
	CACGTGMOTIF					o				1
	GT1MOTIFPSRBCS								o	1
	HEXMOTIFTAH3H4							o		1
	RYREPEATVFLEB4			o						1
	ACGTTBOX									1
	MEJARELELOX							o		1
	SP8BFIBSP8AIB							o		1
	SURE2STPAT21			o						1
	RGATAOS								o	1
	AGCBOXNPGLB									1
	LTRE1HVBLT49		o							1
Light stress	TATCCAYMOTI-FOSRAMY3D						o			1
	PYRIM-IDINEBOXHVEPB1									1
	AGATCONSENSUS					o				1
	AGL1ATCONSENSUS					o				1
	AGL2ATCONSENSUS			o						1
	PROLAMINBOX-OSGLUB1								o	1
	CACGCAATGMGH3									1
	HDZIP2ATATHB2	o								1
	TGACGTVMAMY							o		1
	L1BOXATPDF1			o						1
	ACGTABREMO-TIFA2OSEM					o				1
	CRTDREHVCBF2							o		1
	GARE1OSREP1								o	1
	GARE2OSREP1									1
	TGTCACAC-MCUCUMISIN						o			1
	BP5OSWX	o								1
	GADOWNAT					o				1
	EMHVCHORD									1
	T/GBOXATPIN2	o								1
	SBOXATRBCS					o				1
	ABRERATCAL					o				1
	CMSRE1IBSPOA								o	1

Note: "ο",have at least one motif， blank, none.

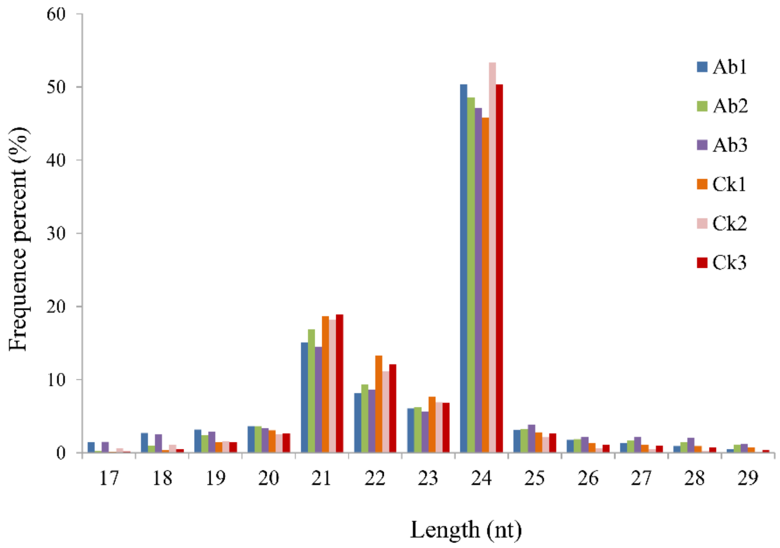

Figure S1 Length distribution of sRNA. Ab, aborting lotus flower buds; Ck, normal lotus flower buds.

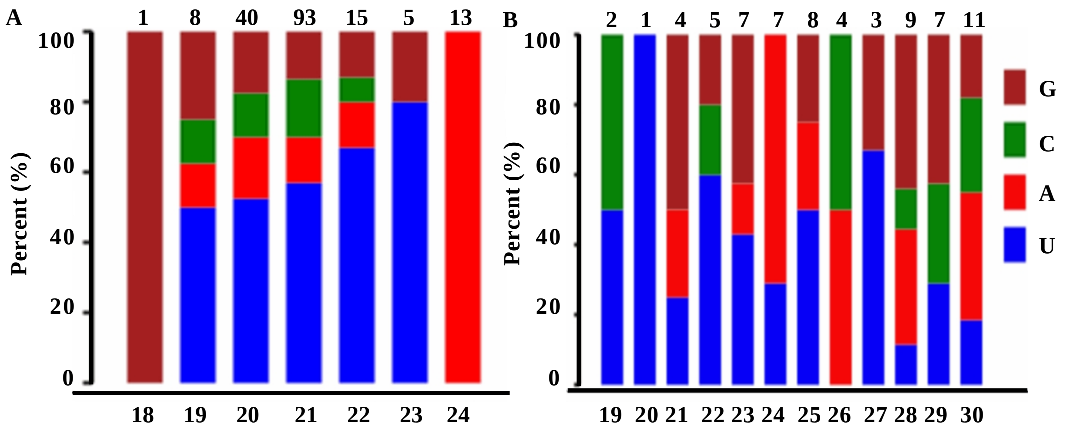

Figure S2 The first base distribution of known and novel miRNAs

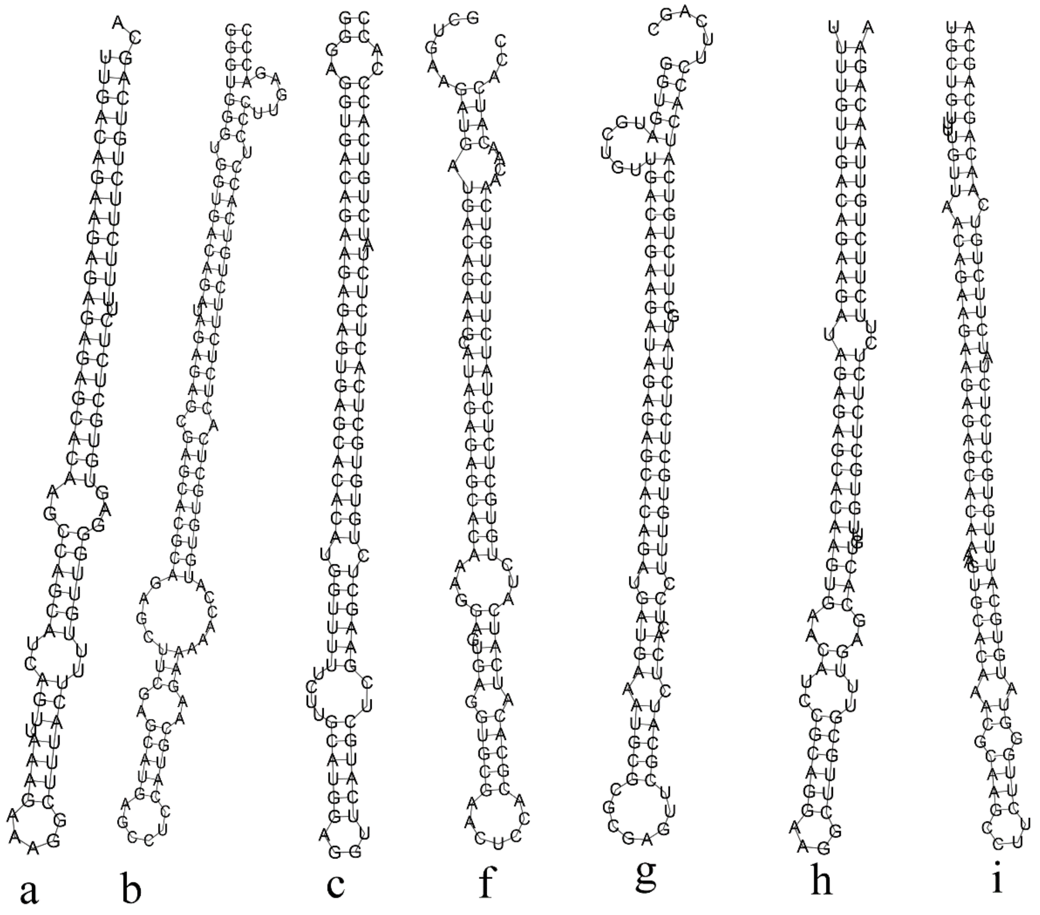

Figure S3 Hairpin structure of miR156 family precursors

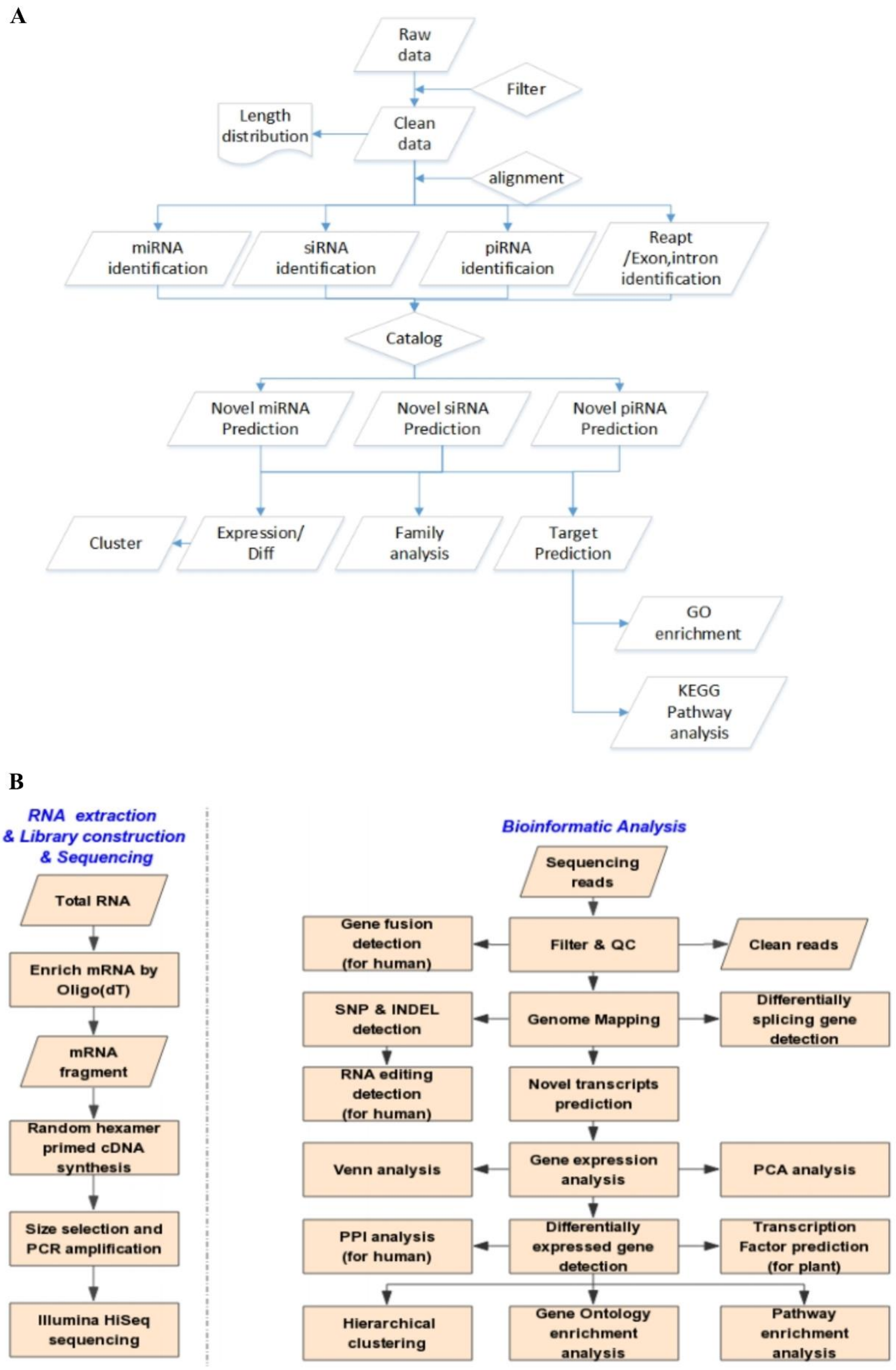

Figure S4 Bioinformatics analysis pipeline for Small RNA (A) and mRNA (B) sequencing.
